# Challenges and Opportunities of Nanotechnology as Delivery Platform for Tocotrienols in Cancer Therapy

**DOI:** 10.3389/fphar.2018.01358

**Published:** 2018-11-26

**Authors:** Geetha Maniam, Chun-Wai Mai, Mohd Zulkefeli, Christine Dufès, Doryn Meam-Yee Tan, Ju-Yen Fu

**Affiliations:** ^1^School of Postgraduate Studies, International Medical University, Bukit Jalil, Malaysia; ^2^Product Development and Advisory Services Division, Malaysian Palm Oil Board, Bandar Baru Bangi, Malaysia; ^3^Department of Pharmaceutical Chemistry, School of Pharmacy, International Medical University, Bukit Jalil, Malaysia; ^4^Centre for Cancer and Stem Cells Research, Institute for Research, Development and Innovation, International Medical University, Bukit Jalil, Malaysia; ^5^Strathclyde Institute of Pharmacy and Biomedical Sciences, University of Strathclyde, Glasgow, United Kingdom; ^6^School of Pharmacy, Monash University Malaysia, Bandar Sunway, Malaysia

**Keywords:** tocotrienols, nanotechnology, drug delivery, cancer, nanoformulation

## Abstract

Plant-derived phytonutrients have emerged as health enhancers. Tocotrienols from the vitamin E family gained high attention in recent years due to their multi-targeted biological properties, including lipid-lowering, neuroprotection, anti-inflammatory, antioxidant, and anticancer effects. Despite well-defined mechanism of action as an anti-cancer agent, their clinical use is hampered by poor pharmacokinetic profile and low oral bioavailability. Delivery systems based on nanotechnology were proven to be advantageous in elevating the delivery of tocotrienols to tumor sites for enhanced efficacy. To date, preclinical development of nanocarriers for tocotrienols include niosomes, lipid nanoemulsions, nanostructured lipid carriers (NLCs) and polymeric nanoparticles. Active targeting was explored via the use of transferrin as targeting ligand in niosomes. *In vitro*, nanocarriers were shown to enhance the anti-proliferative efficacy and cellular uptake of tocotrienols in cancer cells. *In vivo*, improved bioavailability of tocotrienols were reported with NLCs while marked tumor regression was observed with transferrin-targeted niosomes. In this review, the advantages and limitations of each nanocarriers were critically analyzed. Furthermore, a number of key challenges were identified including scale-up production, biological barriers, and toxicity profiles. To overcome these challenges, three research opportunities were highlighted based on rapid advancements in the field of nanomedicine. This review aims to provide a wholesome perspective for tocotrienol nanoformulations in cancer therapy directed toward effective clinical translation.

## Introduction

Plant-derived phytochemicals played a pivotal role in the development of cancer therapeutics. Many anti-cancer agents used in clinical practice are derived from natural phytochemicals (e.g., camptothecin and paclitaxel) but they are highly associated with life-threatening toxicities (Iqbal et al., 2017). As such, recent paradigm shifts in cancer research led to global interest on new phytochemicals as adjuvant therapy such as epigallocatechin-3-gallate, curcumin, vanilloids, flavones, jerantinine, and vitamin E (Lemarié et al., 2012; Chung et al., 2017; Soo et al., 2017; Mai et al., 2018). These natural therapeutics often have lower potency compared to conventional chemotherapeutics but are favored due to their low toxicity profiles.

Vitamin E is a family of compounds consisting two major groups, i.e., tocopherols and tocotrienols. They are differentiated by the amount of saturation on the side chains, each having four homologs (α, β, γ, δ). Although the physiological role of α-tocopherol is well-established, little is known about the nutritional mechanisms of tocotrienols. The anti-cancer properties of tocotrienols were reported in multiple cancer cell lines including breast, lung, liver, pancreatic, and bladder cancers (Shun et al., 2004; Husain et al., 2011). Tocotrienols were reported to target the hallmarks of cancer via pro-apoptotic (caspase-8, Bid, Bax, mitochondrial membrane permeability), modulation of growth factors (EGF-dependent PI3K pathway, TGF-β), cell cycle arrest (cyclin D1, p21, p27), anti-angiogenesis (VEGF, VEGFR-2) and anti-metastatic properties (E-cadherin, MMP-9, EMT, CXCR4) (Samant and Sylvester, 2006; Hanahan and Weinberg, 2011; Nazzal and Sylvester, 2012; Lim et al., 2014; Ye et al., 2015; De Silva et al., 2016; Xun et al., 2017).

Despite active research, global recognition of tocotrienols is still lacking, due to their low abundance in food sources. While tocopherols are mainly found in soy bean and sunflower oil, tocotrienols can be found in palm oil and rice bran oil (Nesaretnam, 2008). Besides, the plasma concentration of palm tocotrienols was detected at a much lower concentration compared to that of tocopherols (Patel et al., 2012), with a short half-life of 4 h (Tasciotti et al., 2008). When investigated in rats, the absolute oral bioavailability of tocotrienols was found to be less than 30% for α-tocotrienol and less than 10% for γ- and δ-tocotrienol (Yap et al., 2003).

One of the strategies to overcome their low oral bioavailability is the application of nanotechnology. Due to their small size and high surface area, nanomedicine exhibit key differences in comparison to bulk materials, which were observed in the context of solubility, pharmacokinetics, efficacy, and toxicity profiles (Ventola, 2017). In this review, we aim to discuss the important similarities and differences among nanoformulations that were investigated on tocotrienols, and hence their challenges and opportunities toward clinical application.

## Application of Nanoformulations for Tocotrienols

**FIGURE 1:**
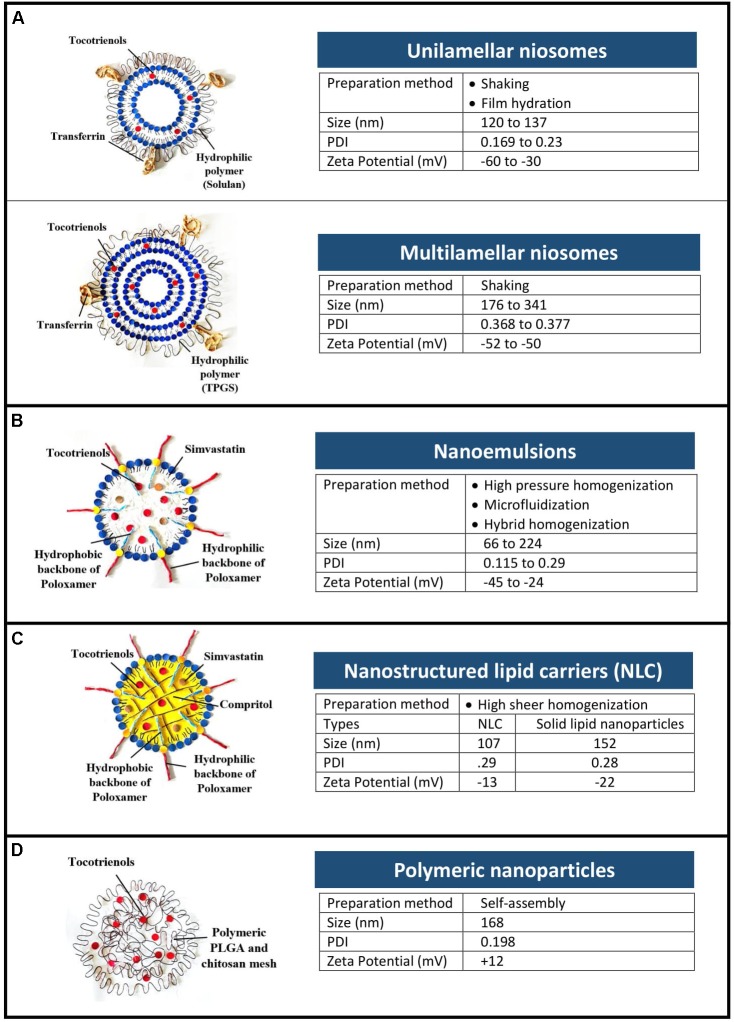
Types and physical characteristics of tocotrienol nanoformulations. **(A)** Niosomes, **(B)** nanoemulsions, **(C)** nanostructured lipid carriers, and **(D)** polymeric nanoparticles.

As shown in Figure 1, four types of nanoformulations had been studied for tocotrienols, i.e., niosomes, nanoemulsions, NLCs, and polymeric nanoparticles.

### Niosomes

Niosomes are bilayer vesicles made of non-ionic surfactants and cholesterol. As depicted in Figure 1A, tocotrienols were entrapped in unilamellar and multilamellar niosomes prepared using Span 60 and AP (Fu et al., 2009; Tan et al., 2017). Multilamellar vesicles were recorded with higher entrapment efficiency (48.6 ± 1.7%) than unilamellar vesicles (28.5 ± 1.9%) owing to the multiple lipid bilayers (Fu et al., 2009, 2011). In cancer cells from human epithelial, brain and ovarian origin, niosomes improved the anti-proliferative activity of tocotrienols by at least 17-fold (Fu et al., 2009, 2011; Karim et al., 2017). In breast cancer cells however, AP-based niosome showed enhanced efficacy of 3.4-fold compared to free tocotrienols (Tan et al., 2017).

In addition, surface modification of niosomes with transferrin was investigated as an active targeting strategy. Being an iron transporter, transferrin was widely investigated as a targeting ligand, able to recognize overexpressed transferrin receptors in various cancer cells (Dufès et al., 2000). *In vivo*, intravenous injection of transferrin-bearing niosomes entrapping tocotrienol led to 60% tumor regression on mice bearing A431 epidermoid carcinoma tumors (Fu et al., 2009). By contrast, tumors from other treatment groups were progressive. This effect was further improved with increasing duration of treatment (from 10 to 20 injections) resulting in 20% complete disappearance of the tumors and 20% tumor regression at the end of the experiment (Fu and Dufès, 2014). Similarly, in B16F10 murine melanoma tumor models, the intravenous administration of tocotrienols entrapped in transferrin-bearing niosomes led to 50% complete disappearance of the tumors (Fu and Dufès, 2014). It was the first time that a tocotrienol formulation was shown to decrease and even fully eradicate a tumor. In a further study, transferrin-bearing multilamellar vesicles entrapping α-tocotrienol led to complete tumor eradication of up to 60% of B16F10 tumors (Karim et al., 2017).

### Nanoemulsions

Nanoemulsions are thermodynamically stable dispersion of two immiscible phase with droplets in the submicron size. Surfactants were selected based on hydrophilic lipophilic balance of the two phases, while method of preparation was chosen among high energy processes that are able to produce stable nanoemulsions. High pressure homogenization (Alayoubi et al., 2013), microfluidization (Goh et al., 2015; Hasan et al., 2018), and PIT emulsification (Pham et al., 2016) were studied, each having distinctive advantages. For example, narrow size distribution was observed in nanoemulsions prepared using microfluidization with increasing pressure and number of cycles (Goh et al., 2015). Hybrid of more than 1 method (e.g., PIT and homogenization) was shown to produce nanoemulsion that remained stable after 2 months’ storage (Pham et al., 2016).

When investigated in skin carcinomas, tocotrienol nanoemulsions reported a higher anti-proliferative effect (IC_50_ in A431 and SCC-4 cells: 43 and 47 μM) compared to tocotrienols/propyleneglycol mixture (IC_50_ in A431 and SCC-4 cells: 217 and 279 μM) (Pham et al., 2016). The anti-proliferative activity of tocotrienol nanoemulsion was also proven in human breast cancer cells (MDA-MB-231 and MCF-7) with IC_50_ of 7 and 14 μM respectively (Alayoubi et al., 2013). Although *in vivo* data is limited, irritancy tests on reconstructed human epidermis and human corneal epithelium models were studied (Hasan et al., 2018). At 5% concentration, nanoemulsions were classified as non-irritants to the eyes and skin according to OECD guidelines.

### Nanostructured Lipid Carriers (NLCs)

Nanostructured lipid carriers are solid colloidal dispersions prepared by mixing a blend of solid and liquid oil matrix (Li et al., 2017). Combination of lipids with low and high melting points creates imperfections in the crystalline lipid core of the NLCs that provide loading capacity for the incorporated compounds (Muller et al., 2002). This property is advantageous for lipophilic compounds such as tocotrienols, where loading capacity was shown in NLC using DSC and NMR techniques (Ali et al., 2010a).

When tested in malignant ++SA cells, tocotrienols-loaded NLCs were 12-fold more efficacious (IC_50_: 1.50 ± 0.12 μM) than α-tocopherol-loaded NLCs (IC_50_: 17.70 ± 0.74 μM) (Ali et al., 2010b). Likewise, the anti-proliferative activities were further enhanced with concurrent delivery of simvastatin/tocotrienols in NLCs (IC_50_: 0.52 ± 0.02 μM). *In vivo*, pharmacokinetic profiles of tocotrienol were investigated in rats after oral administration of lipid nanoparticles at 10 mg/kg body weight (Abuasal et al., 2012). Based on plasma concentrations over 12 h, the AUC and Cmax were approximately three times higher when delivered in lipid nanoparticles compared to mixed micelles. Furthermore, the onset of absorption is faster in lipid nanoparticles, i.e., Tmax of 3 vs. 5 h for mixed micelles.

### Polymeric Nanoparticles

Polymeric nanoparticles are commonly constructed using amphiphilic polymers with two or more polymer chains of varied hydrophobicity, having the ability to form self-assembled micelles in the presence of aqueous solution (Mir et al., 2017). A hybrid system using poly(lactic-co-glycolic) acid (PLGA) and chitosan was prepared by synthesizing PLGA-tocotrienol copolymer (Alqahtani et al., 2015). In breast cancer cells, the cellular uptake of tocotrienols was more than twofold higher when delivered using hybrid nanoparticles compared to mixed micelles. In correlation, the anti-proliferative activity of tocotrienols in MDA-MB-231 cells were enhanced by 25% when encapsulated in hybrid polymeric nanoparticles compared to PLGA nanoparticles.

## Challenges

**FIGURE 2:**
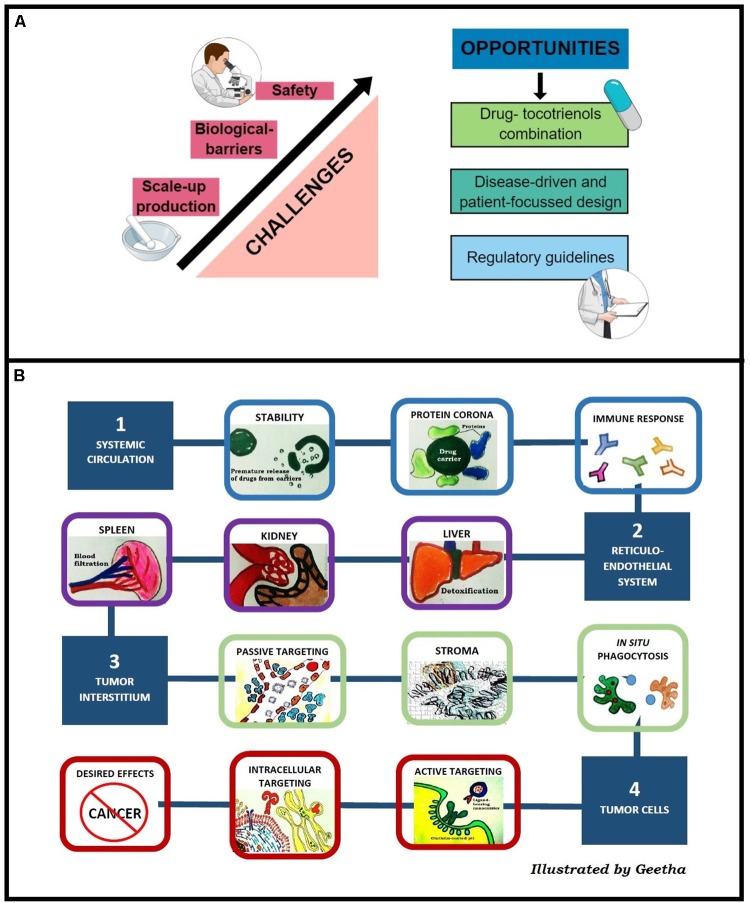
**(A)** Challenges and opportunities of tocotrienol nanocarriers in clinical translation. **(B)** Stumbling blocks of nanocarriers to reach tumors classified into four major obstacles, i.e., stability in systemic circulation, RES, extravasation into tumor interstitium, and intracellular uptake.

From a formulation point of view, enhanced efficacy was observed when tocotrienols were delivered using nanocarriers, irrespective of the types. Nevertheless, the average success rate of a novel therapeutic to travel from Phase 1 trial to regulatory approval is merely 10% (Hay et al., 2014). To bridge the gap, we identified three major challenges for clinical translation of tocotrienol nanocarriers as depicted in Figure 2A.

### Scale-Up Production

Among the tocotrienol nanocarriers reported here, nanoemulsions were prepared using top–down method whereas niosomes, NLC, and polymeric nanoparticles were prepared using bottom–up techniques. For nanoemulsions, high energy processes are required to achieve stability against coalescence, creaming, and Ostwald ripening (Cinar, 2017). In bottom–up methods, nanocarriers were formed by self-assembly of their respective building blocks, controlled by the growth or nucleation of lipids and polymers used (Robertson et al., 2016). Concise control over the growth of building blocks is significantly hindered during scale-up production. Hence, batch to batch variation tends to be high for nanoformulations prepared using bottom–up methods such as silica nanoparticles (diameter ranging from 32 to 160 nm between eight batches) (Mulhopt et al., 2018). In addition, variation in mixing method, time and solvent properties during scale-up production strongly defines the size distribution of nanoformulations (Valencia et al., 2012). Size distribution has a direct correlation with storage stability due to its implication on the rate of agglomeration and sedimentation of particles (Izak-Nau et al., 2015). Among the nanocarriers discussed, freeze-dried technique was used for niosomes and polymeric nanoparticles. Niosomes in freeze-dried powder remained stable retaining more than 85% of encapsulated tocotrienols after 2 months’ storage (Tan et al., 2017). Besides, NLC was reported to be stable for 6 months as dispersion at room temperature, whereas nanoemulsions was stable up to 28 days at 4°C (Nazzal and Sylvester, 2012; Goh et al., 2015).

For efficient scale up production, continuous synthesis systems were explored (Betke and Kickelbick, 2014). In a continuous microfluidic system, reaction kinetics and formulation composition can be controlled via manipulation of the relative flow rate (Lu et al., 2016). In fact, these devices have been used to synthesize organic and inorganic nanoparticles and were thoroughly reviewed in Valencia et al. (2012).

### Biological Barriers

As illustrated in Figure 2B, there is a need for nanocarriers to cross multiple biological barriers before reaching tumors. Prolonged residence time in the systemic circulation is a major challenge for nanomedicine due to the rapid sequestration by macrophages and the RES. Stealth or long circulating nanocarriers can be prepared using hydrophilic polymers. Solulan and TPGS were investigated for niosomes while Poloxamer 188 was investigated for nanoemulsion and NLC. Upon hydration with fluid, a thick polymer coat that forms around the nanocarriers (Kumar and Rajeshwarrao, 2011) creates steric repulsion that minimizes the interaction with macrophages, thus extending the residence time of vesicles in the systemic circulation (Wilhelm et al., 2016).

During vascular extravasation, most tocotrienol nanocarriers exploit the EPR effect or passive targeting. The EPR effect enables nanoparticles to be extravasated from systemic circulation to tumor site through disorganized tumor vasculature and retained in the tumor interstitium owing to poor lymphatic drainage (Bazak et al., 2014). However, clinical translation of passively targeted nanoparticles are hindered by heterogeneity of the EPR effect between and within cancer types. Tumor vascularity does not only differ in cancer types but in primary and metastatic tumors of same type of cancer (Prabhakar et al., 2013). To overcome this limitation, active targeting was reported as a strategic method. To this end, niosome was investigated for active targeting using transferrin as a ligand, showing up to 60% of tumor eradication. Indeed, in a multivariate analysis of 117 studies on nanoparticles tumor delivery, active targeting showed significantly correlation with delivery efficiency compared to passive targeting (Wilhelm et al., 2016).

At the cellular level, although positively-charged nanosystems have higher cellular uptake (Patiño et al., 2015), nanocarriers with negative zeta potential tend to minimize non-specific internalization from negatively charged cell membrane (Karim et al., 2017). Among the tocotrienol nanocarriers, niosomes, nanoemulsions and NLC are negatively-charged, whereas hybrid polymeric nanoparticles are positively-charged. Analysis by Wilhelm et al. (2016) reported a direct relationship between intracellular uptake and nanocarrier surface charge where highest uptake was recorded for carriers with zeta potential above +10 mV. In agreement, chitosan-grafted PLGA nanoparticles bearing zeta potential of 12.2 ± 3.1 mV were shown to accumulate in Caco2 colon cancer cell at 3.5-fold higher compared to naked PLGA particles which are negatively charged (-4.9 ± 1.8 mV) (Alqahtani et al., 2015). In a review by Blanco et al., an ideal system would be one that bears a neutral or slightly negative charge in blood circulation, and functionalized to switch to positive surface charge upon contact with tumor cells to facilitate internalization (Blanco et al., 2015). To achieve this, Yuan et al. (2012) investigated the use of zwitterion polymers that carry reversible surface charges at different pHs.

### Safety

The safety profile of a drug can be dramatically shifted when delivered in nanocarriers. For example, the known cardiotoxicity of doxorubicin was markedly reduced when delivered in liposomes accompanied with an increased incidence of hematological toxicity and palmar–plantar erythrodysesthesia (Lorusso et al., 2007; Rafiyath et al., 2012; Rom et al., 2014). In general, due to preferential accumulation in the RES system, symptoms associated with kidney and liver toxicity are major concerns for nanocarriers. In addition, immunological reaction to nanomedicine, specifically the CARPA has been widely reported. In a comprehensive review, clinical events of CARPA were previously reported in liposomes, micellar, polymer-based and protein-based nanocarriers (Szebeni, 2014). Material selection was shown to be a major determinant for biocompatibility and strongly differentiates the toxicity profile of nanocarriers. Materials such as lipids used in niosomes and NLC are naturally more biocompatible compared to inorganic materials and carbon-based delivery systems (Ventola, 2017). In a nutshell, nanotoxicity is governed by multiple factors where increased complexity of a nanocarrier will inevitably complicate its toxicity profile.

Due to the lack of harmonized protocols in nanotoxicity studies, *in vivo* toxicity data of tocotrienol nanocarriers were largely dependent on examination of animal wellness and behavior (Fu et al., 2009; Karim et al., 2017). In fact, *in vivo* data of tocotrienol nanocarriers to date are fairly limited. For example, pharmacokinetic profiles were only available for NLCs and remain critically in need for nanocarriers (Abuasal et al., 2012). Besides, the percentage of injected dose in tumors has not been quantified in tocotrienols delivered using nanocarriers. To this end, novel development in image-guided studies with labeled-tocotrienols can be exploited.

## Opportunities

Despite significant challenges, more than 50 nanodrugs have received FDA approval and being used in clinical practice (Bobo et al., 2016). A big proportion of these nanopharmaceutics are indicated for cancer, antimicrobial drugs and bone substitutes to name a few (Ventola, 2017). When categorized according to types, liposomes, polymeric nanoparticles and nanocrystals dominated the list, while there is an increasing trend of metallic and micelle-based nanodrugs undergoing clinical trials (Bobo et al., 2016). Among the tocotrienol nanocarriers that we have analyzed in this review, lipid vesicles and polymeric nanoparticles are more familiar to the industry whereas NLC and nanoemulsions are the emerging nanocarriers slowly gaining recognition in the market. Nevertheless, considering the market prospect of nanodrugs valued at USD 178 billion complemented by the estimated market share of natural products valued over USD 200 billion, opportunities lie in the hands of market-driven research (Ventola, 2017). In this section, several research opportunities for tocotrienol nanocarriers are outlined.

### Combination With Chemotherapeutics

To overcome the compromised efficacy of natural products, adjuvant therapy in combination with chemotherapeutics is an attractive strategy. For example, combination of curcumin and docetaxel in breast cancer patients is currently being studied in a phase-2 clinical trial (NCT00852332). It was suggested that combination of phytochemicals with anticancer agents may potentially increase the efficacy of the cancer drugs and reduce the overall toxicity (Er et al., 2018). Specifically, Eitsuka et al. (2016) comprehensively reviewed combination of tocotrienols with chemotherapeutic agents, where synergistic effect was observed when combined with statins, celecoxib, and gefitinib. To ensure consistent concurrent delivery of tocotrienols-drug combinations, drug delivery systems including nanoemulsions and NLC were investigated. In a study with simvastatin and tocotrienols nanoemulsion, lower IC_50_ values in MDA-MB-231 (4.9 μM) and MCF-7 cells (10.3 μM) were observed compared to simvastatin alone in MDA-MB-231 (8 μM) and MCF-7 cells (19 μM) (Alayoubi et al., 2013). Co-loading of tocotrienols and curcumin in nanoemulsion also showed synergistic anticancer effects in human breast (MCF-7) and ovarian (OVCAR-8) cancer cells (Steuber et al., 2016). In NLC, Ali et al. (2010b) reported 65% lower in IC_50_ values with concurrent delivery of simvastatin/tocotrienols than tocotrienols alone in +SA. Indeed, delivery of a consistent ratio of combinatory drugs is crucial to maintain the synergistic effect. In a review by Zhang et al. (2016), the rationale and advantages of various nanocarriers were discussed.

### Disease-Driven and Patient-Focused Design

The potentials of EPR-based nanomedicine are hampered by the complexity of cancer types that practically limits the efficacy. Early understanding on the behavior of nanomedicines in tumors as a disease-derived approach is crucial (Hare et al., 2017). In recent years, computational methods or simulated tools to obtain detailed view of nanocarriers-organs interactions during their transport to tumors have become increasingly popular. Kiew et al. (2017) and Ho et al. (2018) investigated the permeability of nanocarriers *in vitro* using biomimetic microfluidic models. These techniques enabled preliminary assessment on the delivery efficiency of nanocarriers, which can provide a feedback system to formulation studies. The differential levels of a nanodrug present in tumor tissues compared to normal tissues determine its safety profile. In a patient-focused approach, *in vivo* models which are clinically relevant shall be developed to assess the distribution of nanomedicine. For a clinically relevant model, multiple characteristics are highlighted including stromal morphology, tumor architecture and vessel distribution (Hare et al., 2017). Such models can be developed using patient-derived tumor explant models and genetically engineered mouse models (Ruggeri et al., 2014). For nanotherapeutics that are actively-targeted, the effectiveness of targeting ligand depends on the affinity of the ligand toward its receptor and number of receptors expressed in the cancer cells (Fahmy et al., 2005). Therefore, patient selection via receptor pre-screening offers potential advantage in the prediction of treatment outcome.

### Regulatory Guidelines

Based on CMC approach, regulatory measures on a global scale are playing important roles in the control mechanism of nanomedicines (Havel et al., 2016). A number of technical specifications published from ISO nanotechnology technical committee 229 described standardized methods for characterization of nanoformulations. From the industry’s perspective, there was a pressing need on FDA guidelines due to the lack of convergent definition of “nanoscale”. In 2014, FDA published three guidelines on the human use of nanomedicine. Working in synchronicity, the NCL was formed in 2004 and has since conducted studies on more than 300 nanoformulations (Tinkle et al., 2014). Another laboratory in Europe, NCL is offering similar partnerships with scientists and pharmaceutical companies. With these regulations in place, early consultation was made possible where research design can be structured toward regulatory clearance as one of the steps for efficient clinical translation.

## Conclusion

In this review, nanotechnology was proven to be an efficient tool in enhancing the delivery and efficacy of tocotrienols. When delivered in nanoformulations, tocotrienols showed more than 10-fold improvement in their anti-proliferative efficacy while animal studies reported up to 60% of complete tumor disappearance using tumor-targeted nanocarriers. Nevertheless, there are several challenges to overcome for clinical translation including scale-up production, multiple biological barriers and safety characterization. Abiding to the quote by Albert Einstein, “In the middle of every difficulty lies opportunity”. Potential research opportunities highlighted in this review were combination strategy, disease-driven and patient-focused study design as well as emerging regulatory guidelines. Using nanotechnology for effective delivery, tocotrienols can potentially be the next medicine for cancer therapy.

## Author Contributions

GM, DT, and J-YF wrote the manuscript. All authors contributed to manuscript revision, read and approved the submitted version.

## Conflict of Interest Statement

The authors declare that the research was conducted in the absence of any commercial or financial relationships that could be construed as a potential conflict of interest.

## Abbreviations

AP: ascorbyl palmitate
AUC: area under the curve
CARPA: complement activation-related pseudo allergy
Cmax: maximum serum concentration of the administered drug
CMC: chemistry, manufacturing and control
CXCR4: C-X-C chemokine receptor type 4
DSC: differential scanning calorimetry
EGF-dependent PI3K: epidermal growth factor-dependent phosphatidylinositol-3-kinases
EMT: epithelial–mesenchymal transition
EPR: enhanced permeability and systems
FDA: Food and Drug Administration
ISO: International Organizations for Standardization
MMP-9: matrix metallopeptidase-9
NCL: Nanotechnology Characterization Laboratory
NLCs: nanostructured lipid carriers
NMR: nuclear magnetic resonance
OECD: Organization for Economic Co-operation and Development
p21: cyclin-dependent kinase inhibitor 1
p27: cyclin-dependent kinase inhibitor 1b
PIT: phase inversion temperature
PLGA: poly(lactic-co-glycolic) acid
RES: reticuloendothelial system
TGF-β: transforming growth factor-beta
Tmax: time taken to achieve maximum serum concentration of the administered drug
VEGF: vascular endothelial growth factor
VEGFR-2: vascular endothelial growth factor receptor 2.

## References

[B1] Abuasal B. S. Lucas C. Peyton B. Alayoubi A. Nazzal S. Sylvester P. W. (2012). Enhancement of intestinal permeability utilizing solid lipid nanoparticles increases gamma-tocotrienol oral bioavailability. *Lipids* 47 461–469. 10.1007/s11745-012-3655-4 22271424

[B2] Alayoubi A. Y. Anderson J. F. Satyanarayanajois S. D. Sylvester P. W. Nazzal S. (2013). Concurrent delivery of tocotrienols and simvastatin by lipid nanoemulsions potentiates their antitumor activity against human mammary adenocarcinoma cells. *Eur. J. Pharm. Sci.* 48 385–392. 10.1016/j.ejps.2012.12.011 23262057

[B3] Ali H. Sayed K. E. Sylvester P. W. Nazzal S. (2010a). Molecular interaction and localization of tocotrienol-rich fraction (trf) within the matrices of lipid nanoparticles: evidence studies by differential scanning calorimetry (dsc) and proton nuclear magnetic resonance spectroscopy (h-1 nmr). *Colloids Surf. B Biointerfaces* 77 286–297. 10.1016/j.colsurfb 20189780

[B4] Ali H. Shirode A. B. Sylvester P. W. Nazzal S. (2010b). Preparation, characterization, and anticancer effects of simvastatin-tocotrienol lipid nanoparticles. *Int. J. Pharm.* 389 223–231. 10.1016/j.ijpharm.2010.01.018 20123009

[B5] Alqahtani S. Simon L. Astete C. E. Alayoubi A. Sylvester P. W. Nazzal S. (2015). Cellular uptake, antioxidant and antiproliferative activity of entrapped alpha-tocopherol and gamma-tocotrienol in poly (lactic-co-glycolic) acid (plga) and chitosan covered plga nanoparticles (plga-chi). *J. Colloid Interface Sci.* 445 243–251. 10.1016/j.jcis.2014.12.083 25622049

[B6] Bazak R. Houri M. Achy S. E. Hussein W. Refaat T. (2014). Passive targeting of nanoparticles to cancer: a comprehensive review of literature. *Mol. Clin. Oncol.* 2 904–908. 10.1016/j.jcis.2014.12.083 25279172 PMC4179822

[B7] Betke A. Kickelbick G. (2014). Bottom-up, wet chemical technique for the continuous synthesis of inorganic nanoparticles. *Inogranics* 2 1–15. 10.3892/mco.2014.356 25279172 PMC4179822

[B8] Blanco E. Shen H. Ferrari M. (2015). Principles of nanoparticle design for overcoming biological barriers to drug delivery. *Nat. Biotechnol.* 33 941–951. 10.1038/nbt.3330 26348965 PMC4978509

[B9] Bobo D. Robinson K. J. Islam J. Thurecht K. J. Corrie S. R. (2016). Nanoparticle-based medicines: a review of fda-approved materials and clinical trials to date. *Pharm. Res.* 33 2373–2387. 10.1007/s11095-016-1958-5 27299311

[B10] Chung F. F. Tan P. F. Raja V. J. Tan B. S. Lim K. H. Kam T. S. (2017). Jerantinine A induces tumor-specific cell death through modulation of splicing factor 3b subunit 1 (SF3B1). *Sci. Rep.* 7:42504. 10.1038/srep42504 28198434 PMC5309811

[B11] Cinar K. (2017). A review on nanoemulsions: preparation methods and stability. *Trakya Univ. J. Eng. Sci.* 18 73–83. 10.1038/srep42504 28198434 PMC5309811

[B12] De Silva L. Chuah L. H. Meganathan P. Fu J. Y. (2016). Tocotrienol and cancer metastasis. *Biofactors* 42 149–162. 10.1002/biof.1259 26948691

[B13] Dufès C. Schatzlein A. G. Tetley L. Gray A. I. Watson D. G. Oliver J. C. (2000). Niosomes and polymeric chitosan based vesicles bearing transferrin and glucose ligands for drug targeting. *Pharm. Res.* 17 1250–1258. 10.1023/A:1026422915326 11145231

[B14] Eitsuka T. Tatewaki N. Nishida H. Nakagawa K. Miyazawa T. (2016). Synergistic anticancer effect of tocotrienol combined with chemotherapeutic agents or dietary components: a review. *Int. J. Mol. Sci.* 17:E1605. 10.3390/ijms17101605 27669218 PMC5085638

[B15] Er J. L. Goh P. N. Lee C. Y. Tan Y. J. Hii L. Mai C. W. (2018). Identification of inhibitors synergizing gemcitabine sensitivity in the squamous subtype of pancreatic ductal adenocarcinoma (PDAC). *Apoptosis* 10.1007/s10495-018-1459-6 [Epub ahead of print]. 29740790

[B16] Fahmy T. M. Fong P. M. Goyal A. Saltzman W. M. (2005). Targeted for drug delivery. *Nano Today* 8(Suppl. 8), 18–26. 10.1016/S1369-7021(05)71033-6

[B17] Fu J. Y. Blatchford D. R. Tetley L. Dufès C. (2009). Tumor regression after systemic administration of tocotrienol entrapped in tumor-targeted vesicles. *J. Control. Release* 140 95–99. 10.1016/j.jconrel.2009.08.017 19709637

[B18] Fu J. Y. Dufès C. (2014). Anti-cancer efficacy of intravenously administered tumor-targeted vesicles entrapping tocotrienol. *Pharm. Nanotechnol.* 2 172–181. 10.2174/2211738503666150119231232

[B19] Fu J. Y. Zhang W. Blatchford D. R. Tetley L. McConnell G. Dufès C. (2011). Novel tocotrienol-entrapping vesicles can eradicate solid tumors after intravenous administration. *J. Control. Release* 154 20–26. 10.1016/j.jconrel.2011.04.015 21539872

[B20] Goh P. S. Ng M. H. Choo Y. M. Boyce A. N. Chuah C. H. (2015). Production of nanoemulsions from palm-based tocotrienol rich fraction by microfluidization. *Molecules* 20 19936–19946. 10.3390/molecules201119666 26556328 PMC6331996

[B21] Hanahan D. Weinberg R. A. (2011). Hallmarks of cancer: the next generation. *Cells* 144 646–674. 10.1016/j.cell.2011.02.013 21376230

[B22] Hare J. I. Lammers T. Ashford M. B. Puri S. Storm G. Barry S. T. (2017). Challenges and strategies in anti-cancer nanomedicine development: an industry perspective. *Adv. Drug Deliv. Rev.* 108 25–38. 10.1016/j.addr.2016.04.025 27137110

[B23] Hasan Z. A. A. Idris Z. Gani S. S. A. Basri M. (2018). *In vitro* safety evaluation of palm tocotrienol-rich fraction nanoemulsions for topical application. *J. Oil Palm Res.* 30 150–162.

[B24] Havel H. Finch G. Strode P. Wolfgang M. Zale S. Bobe I. (2016). Nanomedicines: from bench to bedside and beyond. *AAPS J.* 18 1373–1378. 10.1208/s12248-016-9961-7 27480318

[B25] Hay M. Thomas D. W. Craighead J. L. Economides C. Rosenthal J. (2014). Clinical development success rates for investigational drugs. *Nat. Biotechnol.* 32 40–51. 10.1038/nbt.2786 24406927

[B26] Ho Y. T. Lee S. W. L. Azman N. A. Loh F. W. Y. Thien N. P. Kah J. C. Y. (2018). Quantifying vascular distribution and adhesion of nanoparticles with protein corona in microflow. *Langmuir* 34 3731–3741. 10.1021/acs.langmuir.8b00322 29502417

[B27] Husain K. Francois R. A. Yamauchi T. Perez M. Sebti S. M. Malafa M. P. (2011). Vitamin E δ-tocotrienol augments the antitumor activity of gemcitabine and suppresses constitutive Nf-kb activation in pancreatic cancer. *Mol. Cancer Ther.* 10 2363–2372. 10.1158/1535-7163.MCT-11-0424 21971120 PMC3237822

[B28] Iqbal J. Abbasi B. A. Mahmood T. Kanwal S. Ali B. Shah S. A. (2017). Plant-derived anticancer agents: a green anticancer approach. *Asian Pac. J. Trop. Biomed.* 7 1129–1150. 10.1016/j.biopha.2018.04.113 29864953

[B29] Izak-Nau E. Huk A. Reidy B. Uggerud H. Vadset M. Eiden S. (2015). Impact of storage conditions and storage time on silver nanoparticles’ physicochemical properties and implications for their biological effects. *RSC Adv.* 5:84172 10.1039/C5RA10187E

[B30] Karim R. Somani S. Robaian M. A. Mullin M. Amor R. McConnell G. (2017). Tumor regression after intravenous administration of targeted vesicles entrapping the vitamin E α-tocotrienol. *J. Control. Release* 246 79–87. 10.1016/j.jconrel.2016.12.014 27993600

[B31] Kiew S. F. Ho Y. T. Kiew L. Kah J. Lee H. B. Imae T. (2017). Preparation and characterization of an amylase-triggered dextrin-linked graphene oxide anticancer drug nanocarrier and its vascular permeability. *Int. J. Pharm.* 534 297–307. 10.1016/j.ijpharm.2017.10.045 29080707

[B32] Kumar G. P. Rajeshwarrao P. (2011). Nonionic surfactant vesicular systems for effective drug delivery—an overview. *Acta Pharm. Sin. B* 1 208–219. 10.1016/j.apsb.2011.09.002

[B33] Lemarié F. Chang C. W. Blatchrofd D. R. Amor R. Norris G. Tetley L. (2012). Antitumor activity of the tea polyphenol epigallocatechin-3-gallate encapsulated in targeted vesicles after intravenous administration. *Nanomedicine* 8 181–192. 10.2217/nnm.12.83 22891867

[B34] Li Q. Cai T. Huang Y. Xia X. Cole S. P. C. Cai Y. (2017). A review of the structure, preparation, and application of NLCS, PNPS, and PLNS. *Nanomaterials* 7 1–25. 10.3390/nano7060122 28554993 PMC5485769

[B35] Lim S. W. Loh H. S. Ting K. N. Bradshaw T. D. Zeenathul N. A. (2014). Cytotoxicity and apoptotic activities of alpha-, gamma- and delta-tocotrienol isomers on human cancer cells. *BMC Complement. Altern. Med.* 14:469. 10.1186/1472-6882-14-469 25480449 PMC4295404

[B36] Lorusso D. Stefano A. D. Carone V. Fagotti A. Pisconti S. Scambia G. (2007). Pegylated liposomal doxorubicin-related palmar-plantar erythrodysesthesia (’hand-foot’ syndrome). *Ann. Oncol.* 18 1159–1164. 10.1093/annonc/mdl477 17229768

[B37] Lu M. Ozcelik A. Grigsby C. L. Zhao Y. Guo F. Leong K. W. (2016). Microfluidic hydrodynamic focusing for synthesis of nanoparticles. *Nano Today* 11 778–792. 10.1016/j.nantod.2016.10.006 30337950 PMC6191180

[B38] Mai C. Kang Y. Nadarajah V. D. Hamzah A. S. Pichika M. R. (2018). Drug-like dietary vanilloids induce anticancer activity through proliferation inhibition and regulation of bcl-related apoptotic proteins. *Phytother. Res.* 32 1108–1118. 10.1002/ptr.6051 29464796

[B39] Mir M. Ahmed N. Rehman A. U. (2017). Recent applications of plga based nanostructures in drug delivery. *Colloids Surf. B Biointerfaces* 159 217–231. 10.1016/j.colsurfb.2017.07.038 28797972

[B40] Mulhopt S. Diabate S. Dilger M. Adelhelm C. Anderlohr C. Bergfeldt T. (2018). Characterization of nanoparticle batch-to-batch variability. *Nanomaterials* 8:E311. 10.3390/nano8050311 29738461 PMC5977325

[B41] Muller R. H. Radtke M. Wissing S. A. (2002). Nanostructured lipid matrices for improved microencapsulation of drugs. *Int. J. Pharm.* 242 121–128. 10.1016/S0378-5173(02)00180-1 12176234

[B42] Nazzal S. Sylvester P. W. (2012). “Nanomedicine and cancer,” in *Tocotrienol Loaded Lipid Nanoparticles in Cancer*, eds Srirajaskanthan R. Preedy V. R. (Boca Raton, FL: CRC Press), 63–83.

[B43] Nesaretnam K. (2008). Multitargeted therapy of cancer by tocotrienols. *Cancer Lett.* 269 388–395. 10.1016/j.canlet.2008.03.063 18504069

[B44] Patel V. Rink C. Gordillo G. M. Khanna S. Gnyawali U. Roy S. (2012). Oral tocotrienols are transported to human tissues and delay the progression of the model for end-stage liver disease score in patients. *J. Nutr.* 142 513–519. 10.3945/jn.111.151902 22298568 PMC3278267

[B45] Patiño T. Soriano J. Barrios L. Ibáñez E. Nogués C. (2015). Surface modification of microparticles causes differential uptake responses in normal and tumoral human breast epithelial cells. *Sci. Rep.* 5:11371. 10.1038/srep11371 26068810 PMC5155550

[B46] Pham J. Nayel A. Hoang C. Elbayoumi T. (2016). Enhanced effectiveness of tocotrienol-based nano-emulsified system for topical delivery system against skin carcinomas. *Drug Deliv.* 23 1514–1524. 10.3109/10717544.2014.966925 25293973

[B47] Prabhakar U. Maeda H. Jain K. Sevick-Muraca E. M. Zamboni W. Farokhzad O. C. (2013). Challenges and key considerations of the enhanced permeability and retention (epr) effect for nanomedicine drug delivery in oncology. *Cancer Res.* 73 2412–2417. 10.1158/0008-5472.CAN-12-4561 23423979 PMC3916009

[B48] Rafiyath S. M. Rasul M. Lee B. Wei G. Lamba G. Liu D. (2012). Comparison of safety and toxicity of liposomal doxorubicin vs. conventional anthracyclines: a meta-analysis. *Exp. Hematol. Oncol.* 1:10. 10.1186/2162-3619-1-10 23210520 PMC3514106

[B49] Robertson J. D. Rizello L. Avila-Olias M. Gaitzsch J. Contini C. Magon M. S. (2016). Purification of nanoparticles by size and shape. *Sci. Rep.* 8:27494. 10.1038/srep27494 27271538 PMC4897710

[B50] Rom J. Bechstein S. Domschke C. Golatta M. Mayer C. Heil J. (2014). Efficacy and toxicity profile of pegylated liposomal doxorubicin (Caelyx) in patients with advanced breast cancer. *Anticancer Drugs* 25 219–224. 10.1097/CAD.0000000000000037 24247203

[B51] Ruggeri B. A. Camp F. Miknyoczki S. (2014). Animal models of disease: pre-clinical animal models of cancer and their applications and utility in drug discovery. *Biochem. Pharmacol.* 87 150–161. 10.1016/j.bcp.2013.06.020 23817077

[B52] Samant G. V. Sylvester P. W. (2006). Gamma-tocotrienol inhibits ErbB3-dependent PI3K/Akt mitogenic signalling in neoplastic mammary epithelial cells. *Cell Prolif.* 39 563–574. 10.1111/j.1365-2184.2006.00412.x 17109639 PMC6496702

[B53] Shun M. C. Yu W. Gapor A. Parsons R. Atkinson J. Sanders B. G. (2004). Pro-apoptotic mechanisms of action of a novel vitamin E analog (α-tea) and a naturally occurring form of vitamin E (δ-tocotrienol) in MDA-MB-435 human breast cancer cells. *Nutr. Cancer* 48 95–105. 10.1207/s15327914nc4801_13 15203383

[B54] Soo H. Chung F. F. Lim K. Yap V. A. Bradshaw T. D. Hii L. (2017). Cudraflavone c induces tumor-specific apoptosis in colorectal cancer cells through inhibition of the phosphoinositide 3-kinase (pi3k)-akt pathway. *PLoS One* 12:e0170551. 10.1371/journal.pone.0170551 28107519 PMC5249192

[B55] Steuber N. Vo K. Wadhwa R. Birch J. Iacoban P. Chavez P. (2016). Tocotrienol nanoemulsion platform of curcumin elicit elevated apoptosis and augmentation of anticancer efficacy against breast and ovarian carcinomas. *Int. J. Mol. Sci.* 17:E1792. 10.3390/ijms17111792 27792193 PMC5133793

[B56] Szebeni J. (2014). Complement activation-related pseudoallergy: a stress reaction in blood triggered by nanomedicines and biologicals. *Mol. Immunol.* 61 163–173. 10.1016/j.molimm.2014.06.038 25124145

[B57] Tan D. M. Fu J. Wong F. Er H. Chen Y. Nesaretnam K. (2017). Tumor regression and modulation of gene expression via tumor-targeted tocotrienol niosomes. *Nanomedicine* 12 2487–2502. 10.2217/nnm-2017-0182 28972460

[B58] Tasciotti E. Liu X. Bhavane R. Plant K. Leonard A. D. Price K. (2008). Mesoporous silicon particles as a multistage delivery system for imaging and therapeutic applications. *Nat. Nanotechnol.* 3 151–157. 10.1038/nnano.2008.34 18654487

[B59] Tinkle S. Mcneil S. E. Muhlebach S. Bawa R. Borchard G. Barenholz Y. C. (2014). Nanomedicines: addressing the scientific and regulatory gap. *Ann. N. Y. Acad. Sci.* 1313 35–56. 10.1111/nyas.12403 24673240

[B60] Valencia P. M. Farokhzad O. C. Karnik R. Langer R. (2012). Microfluidics technologies for accelerating the clinical translation of nanoparticles. *Nat. Nanotechnol.* 7 623–629. 10.1038/nnano.2012.168 23042546 PMC3654404

[B61] Ventola L. (2017). Progress in nanomedicine: approved and investigational nanodrugs. *P T* 42 742–755. 29234213 PMC5720487

[B62] Wilhelm S. Tavares A. J. Dai Q. Ohta S. Audet J. Dvorak H. F. (2016). Analysis of nanoparticle delivery to *tumors*. *Nat. Rev. Mater.* 1 1–11. 10.1038/natrevmats.2016.14

[B63] Xun C. Mamat M. Guo H. Mamati P. Sheng J. Zhang J. (2017). Tocotrienol alleviates inflammation and oxidative stress in a rat model of spinal cord injury via suppression of transforming growth factor-beta. *Exp. Ther. Med.* 14 431–438. 10.3892/etm.2017.4505 28672950 PMC5488605

[B64] Yap S. P. Yuen K. H. Lim A. B. (2003). Influence of route of administration on the absorption and disposition of alpha-, gamma- and delta-tocotrienols in rats. *J. Pharm. Pharmacol.* 55 53–58. 10.1111/j.2042-7158.2003.tb02433.x 12625867

[B65] Ye C. Zhao W. Li M. Zhuang J. Yan X. Lu Q. (2015). Delta-tocotrienol induces human bladder cancer cell growth arrest, apoptosis and chemosensitization through inhibition of STAT3 pathway. *PLoS One* 10:e0122712. 10.1371/journal.pone.0122712 25849286 PMC4388509

[B66] Yuan Y. Y. Mao C. Q. Du X. J. Du J. Z. Wang F. Wang J. (2012). Surface charge switchable nanoparticles based on zwitterionic polymer for enhanced drug delivery to tumor. *Adv. Mater.* 24 5476–5480. 10.1002/adma.201202296 22886872

[B67] Zhang R. X. Wong H. L. Xue H. Y. Eoh J. Y. Wu X. Y. (2016). Nanomedicine of synergistic drug combinations for cancer therapy - Strategies and perspectives. *J. Control. Release* 240 489–503. 10.1016/j.jconrel.2016.06.012 27287891 PMC5064882

